# The Role of Different Alkali Metals in the A_15_Tl_27_ Type Structure and the Synthesis and X-ray Structure Analysis of a New Substitutional Variant Cs_14.53_Tl_28.4_

**DOI:** 10.3390/ma14247512

**Published:** 2021-12-08

**Authors:** Vanessa F. Schwinghammer, Susanne M. Tiefenthaler, Stefanie Gärtner

**Affiliations:** 1 Institute of Inorganic Chemistry, University of Regensburg, 93040 Regensburg, Germany; Vanessa.Schwinghammer@ur.de (V.F.S.); Susanne.Tiefenthaler@ur.de (S.M.T.); 2 Central Analytics, X-ray Crystallography Department, University of Regensburg, 93040 Regensburg, Germany

**Keywords:** thallide, intermetallics, single crystal, X-ray structure analysis

## Abstract

Alkali metal thallides have been known since the report of E. Zintl on NaTl in 1932. Subsequently, binary and ternary thallides of alkali metals have been characterized. At an alkali metal proportion of approximately 33% (A:Tl~1:2, A = alkali metal), three different unique type structures are reported: K_49_Tl_108_, Rb_17_Tl_41_ and A_15_Tl_27_ (A = Rb, Cs). Whereas Rb_17_Tl_41_ and K_49_Tl_108_ feature a three-dimensional sublattice of Tl atoms, the A_15_Tl_27_ structure type includes isolated Tl_11_ clusters as well as two-dimensional Tl-layers. This unique arrangement is only known so far when the heavier alkali metals Rb and Cs are included. In our contribution, we present single-crystal X-ray structure analyses of new ternary and quaternary compounds of the A_15_Tl_27_ type structure, which include different amounts of potassium. The crystal structures allow for the discussion of the favored alkali metal for each of the four Wyckoff positions and clearly demonstrate alkali metal dependent site preferences. Thereby, the compound Cs_2.27_K_12.73_Tl_27_ unambiguously proves the possibility of a potassium-rich A_15_Tl_27_ phase, even though a small amount of cesium appears to be needed for the stabilization of the latter structure type. Furthermore, we also present two compounds that show an embedding of Tl instead of alkali metal into the two-dimensional substructure, being equivalent to the formal oxidation of the latter. Cs_14.53_Tl_28.4_ represents the binary compound with the so far largest proportion of incorporated Tl in the structure type A_15_Tl_27_.

## 1. Introduction

Alkali metal thallides represent a very interesting class of materials in terms of structural chemistry as they involve versatile thallium substructures depending on the amount of alkali metal involved, which is equitable to the valence electron concentration [1]. The electronic description of the latter compounds is not trivial, as part of them are diamagnetic and show a real band gap; therefore, the description by the Zintl-Klemm formalism is permissible in a narrow sense [2,3,4,5]. In contrast, quite a large number of these compounds show metallic and paramagnetic behavior [6,7] and the basis of this concept, the complete electron transfer from the electropositive to the electronegative element, is not true for these materials. Nevertheless, in many cases, the formed anionic partial structures can be described according to this theory [8,9,10]. This still makes this concept a very powerful tool in solid state chemistry at the frontier between metallic and ionic bonding. The first Zintl phase goes back to the investigations of E. Zintl, who described the crystal structure of NaTl, in which the thallium substructure follows the Zintl–Klemm formalism by forming a diamond sublattice [11]. Interestingly, thallium as a “parental” element for this concept is found left to the so-called Zintl border in the periodic table of elements [12,13]. This actually makes it a very interesting element to point out and describe structural effects of the rather innocent electropositive counterpart on structure formation, as it can be seen as element “at the boundary of the border”. Alkali metal thallides experienced a renaissance due to the investigations of *J. D. Corbett* in the 1990′s [8,9]. One of the type structures he reported on in 1996 is A_15_Tl_27_, which could be realized for the heavier congeners rubidium and cesium [14]. The crystal structures of these compounds showed the involvement of two thallium substructures. On the one hand, discrete Tl_11_^7—^ clusters are present, which are also known from binary A_8_Tl_11_ (A = K, Rb, Cs) phases crystallizing in the K_8_In_11_-type structure [7,15]. In these compounds, an extra electron is present A_8_[Tr_11_^7—^][e^—^] being responsible for pauli paramagnetism and metallic behavior. This extra electron can be replaced by halide in A_8_Tr_11_X, and for Cs_8_Ga_11_Cl diamagnetic behavior is observed [16,17,18,19]. Detailed investigations on A_8_Tl_11_X showed that this replacement is accompanied by a less pronounced distortion of the Tl_11_ clusters from ideal D_3*h*_ symmetry [17,18]. In general, Tl_11_^7—^ clusters represent a favored geometry as they also can be perceived as double-tetrahedral star units, which represent a very common structural motif in intermetallic compounds [20]. For example, in K_18_Tl_20_Au_3_ as molecular building blocks also Tl_11_^7—^ cluster anions and additional Tl_9_Au_2_^9—^ polyanions are present [21]. Due to the high symmetry of the hexagonal crystal structure of the latter, the point group of the Tl_11_ cluster in this case is D_3*h*_. In the here investigated A_15_Tl_27_ compounds, beside the Tl_11_ clusters, an additional subunit of thallium atoms is present, two-dimensional Tl_16_^8—^ layers, which include large pores, in which alkali metal atoms reside [14]. In the compound Rb_14_CsTl_27_, the preference of the larger alkali metal cesium residing in the pore was proven. A related compound to the A_15_Tl_27_ type structure was reported in 1997 for K_14_Cd_9_Tl_21_. Here, one symmetry inequivalent thallium site of the layer was replaced by one cadmium atom. Additionally, the alkali metal position being located in the pore could be substituted by a Cd_3_ triangle, which gave rise to the formation of two-dimensional [Cd_9_Tl_10_]^7—^ layers [22]. Band structure and extended Hückel calculations indicated a two-dimensional metal for the latter compound. Whereas in K_14_Cd_9_Tl_21_ potassium acts as counter cation, binary K_15_Tl_27_ has not yet been reported. In contrast, for binary thallides with an alkali metal content of appr. 0.3, for potassium only K_49_Tl_108_ is reported [23]. This compound includes a three-dimensional thallium network. The change from potassium to larger, more electropositive alkali metals accounts for a change in the type of structure accompanied by a significant change in the thallium sublattice. A similar dependency on the alkali metal involved has previously been reported for ATl (A=Li-Cs) [8,9,11,24,25,26,27,28]. In the region of A:Tl (1:2) the absence of K_15_Tl_27_ [14] on the one hand and on the other hand the observation of K_49_Tl_108_ [23,29] makes compositions, including different alkali metals, very promising candidates for the investigations of the influence of the latter on structure formation. In the search of a composition near K_15_Tl_27_, we here report on different ternary and a quaternary A_15_Tl_27_ phases, including potassium. The influence of the mixed alkali metals on the thallium substructure is discussed. Additionally, the compounds Cs_14.53_Tl_28.4_ and Cs_8.21_Rb_6.76_Tl_27.09_ give the first evidence, that replacement of the alkali metal in the pores of the two-dimensional layer in Cs_15_Tl_27_ by thallium is possible.

## 2. Materials and Methods

All compounds have been prepared by using classical solid-state techniques. The alkali metals cesium and rubidium were obtained by reduction in the alkali metal chlorides with elemental calcium [30] and afterwards distilled twice for purification. Potassium was segregated for purification. Thallium drops (ABCR, purity 99.99%) were used without further purification and were stored under inert gas atmosphere. The starting materials were placed in tantalum ampoules and sealed in argon atmosphere. The sealed crucibles were afterwards placed in quartz glass tubes (QSIL GmbH, Ilmenau, Germany) and sealed under argon atmosphere. The same temperature program was used for all compounds: heating up to 973.15 K with a heating rate of 100 K/h, holding for 24 h, cooling to room temperature with a cooling rate of 3 K/h.

The received products are very sensitive towards moisture and oxygen; therefore, they were stored in a glove box (Labmaster 130 G Fa. M. Braun, Garching, Germany). In advance of the characterization by single crystal X-ray diffraction techniques, a small number of crystals was transferred into dried mineral oil. Subsequently, a suitable crystal was isolated and mounted on the Rigaku SuperNova diffractometer (Rigaku Polska Sp. Z o. o. Ul, Wroclaw, Poland) (X-ray: Mo-source, Eos detector) using MiTeGen loops, before collecting data at 123 K. The program CrysAlisPro was used for data collection and data reduction [31]. The solution of the structure and subsequent refinements were accomplished in Olex2 [32] using ShelXT [33,34]. For generating representations of the crystal structures, the software Diamond was used [35].

Powder diffraction samples were prepared in sealed capillaries (ø 0.3 mm, WJM-Glas-Müller GmbH, Berlin, Germany) and data collection was performed on a STOE Stadi P diffractometer (STOE, Darmstadt, Germany) (monochromatic Mo-K_α1_ radiation λ = 0.70926 Å) equipped with a Dectris Mythen 1 K detector. The visualization and indexation was carried out by the software WinXPOW [36].

## 3. Results

All compounds crystallize in the A_15_Tl_27_ (A = Cs, Rb)-type structure (hexagonal, space group *P*-62*m*) [14]. These alkali metal thallides naturally possess very high absorption coefficients (MoKα, µ > 70 mm^−1^), hence small single crystals were selected for the X-ray diffraction experiments, but the data sets still suffered from severe absorption effects, which could be reduced by applying absorption correction. The high redundancy of the collected data sets additionally allowed a shape adjustment by the “shape optimization” tool in the CrysAlisPro software (diffractometer software, Rigaku).

We first observed the evidence of the mixed A_15_Tl_27_ phases (K_6.96_Rb_8.04_Tl_27_ and Cs_8.21_Rb_6.76_Tl_27.09_) during our studies concerning A_8_T_11_X compounds, where they crystallized as a by-product together with K_3.98_Rb_4.02_Tl_11_Cl_0.1_ and Cs_5.13_Rb_2.87_Tl_11_Cl_0.49_, respectively. The smaller amount of incorporated chloride in these compounds, which simultaneously means a higher degree of reduction, facilitated the formation of the less reduced A_15_Tl_27_ phases, which include less alkali metal per Tl. As the results of these crystal structure determinations allowed deeper insights in the alkali metal dependent site preferences, we subsequently started to prepare and characterize mixed alkali metal approaches following the composition A_15_Tl_27_ (A=K, Rb, Cs). Table 1 shows the data of the structure determinations of all approaches, including potassium and at least one other alkali metal.

For the ternary approaches including potassium, we additionally obtained the less reduced A_49_Tl_108_ phases as side products [15,23]. This is reasonable, as the amount of alkali metal is very similar in both compounds (0.357 for A_15_Tl_27_ and 0.312 for K_49_Tl_108_). Additionally, we observed formations of multicrystals between the A_15_Tl_27_ and A_49_Tl_108_ phases. This might be due to the fact that the longest side of the unit cell of the A_15_Tl_27_ and the cell vector of the A_49_Tl_108_ (cubic, *Pm*-3, a~17Å) are close in length.

Cs_8.21_Rb_6.76_Tl_27.09_ exhibits residual electron density near the alkali metal position A4 (d(A4-q) > 2.7 Å), for which the assignment of Tl is reasonable in terms of the observed Tl-Tl distances (see Section 4.3 Discussion). However, the obtained s.o.f. of less than 10% thallium made this interpretation suspicious; therefore, binary samples involving cesium and a higher amount of thallium were prepared which all led to the composition Cs_14.53_Tl_28.4_. *Corbett*’s Cs_15_Tl_27_ was prepared in addition and the absence of additional thallium in this phase was confirmed. Table 2 gives the data of the structure solution and refinement of Cs_8.21_Rb_6.76_Tl_27.09,_ Cs_14.53_Tl_28.4_ and Cs_15_Tl_27_.

The refinement of Cs_14.53_Tl_28.4_ undoubtedly proves the presence of thallium beside a less occupied alkali metal position (s.o.f. 0.527(5)) which complements unity by an additional Tl site (s.o.f. 0.473(5)). Any attempt to incorporate more Tl, including a fully occupied thallium site, which would be equivalent to Cs_14_Tl_30_, always led to the composition of Cs_14.53_Tl_28.4_.

Powder diffraction patterns of Cs_2.27_K_12.73_Tl_27_ and Cs_14.5_Tl_28.4_ were recorded (indexing and diffraction patterns see Figures S1–S3, Supplementary Material). In general, the approaches involving potassium additionally produced A_49_Tl_l08_ beside A_15_Tl_27_, which could be observed in the powder diffraction patterns.

Cs_15_Tl_27_ and Cs_14.53_Tl_28.4_ naturally show very similar diffraction patterns. The indexed unit cell parameters according to the powder diffraction patterns refined to the values a = 10.8580(19) Å and c = 18.108(3) Å (Cs_14.5_Tl_28.4_) and a = 10.501(5) Å and c = 18.157(5) Å (Cs_15_Tl_27_). This confirms the trend of the unit cell parameters obtained for the single crystals. The deviations between the unit cell parameters of Cs_15_Tl_27_ and Cs_14.5_Tl_28.4_, respectively, each derived from single crystal and powder diffraction data, are due to the different applied temperatures (single crystal: 123 K; powder diffraction: room temperature (298 K)).

## 4. Discussion

### 4.1. Occupation Trends of the Alkali Metal Positions

**Figure 1:**
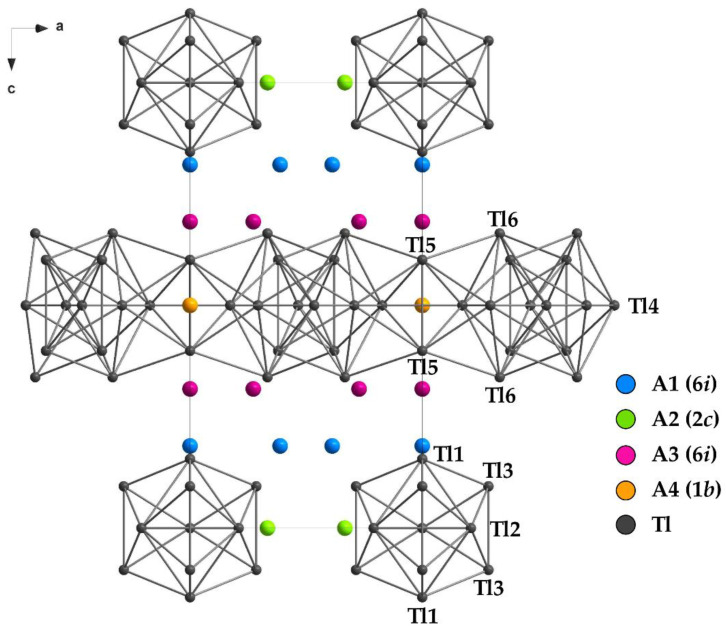
Unit cell of the A_15_Tl_27_ type structure with the 4 symmetry-independent alkali metal positions.

During our search for binary K_15_Tl_27_ and the role of the different alkali metal sites in A_15_Tl_27_ in general, different ternary and quaternary approaches were applied. Those new compounds gave insight into the site preferences of the different alkali metals. In A_15_Tl_27_, four different alkali metal sites are present (see Figure 1). Corbett et al. showed for Rb_14_CsTl_27_, that cesium preferably resides at position A4, as the rather large pore within the two-dimensional layer allows more space for the larger alkali metal [14]. By having a closer look at the surroundings of the remaining alkali metals, additional site preferences would be conceivable [1]. In further detail, the alkali metal positions A1 and A3 are distinguished from A2 and A4 by their number and distances of surrounding atoms, which is also reflected in their crystallographic site symmetry (see Figure 1). Alkali metals on the Wyckoff-position 6*i* separate the Tl-layer from the isolated Tl_11_ clusters. Here, less contacts within a smaller range of distances are observed, whereas alkali metal atoms on position 1*b* (within the pores of the Tl-layer) and 2*c* (between the isolated clusters) show larger distances to a higher number of neighboring atoms [1,14]. As the positions A2 and A4 show contacts to a larger number of neighboring atoms and additionally more space around them is available, they are likely to be preferable positions for the heavier alkali metals. This assumption can be reinforced by the new ternary and quaternary compounds (Table 3). The distances from alkali metal to thallium atoms naturally increases with increasing size of the alkali metal. This trend is also reflected in the unit cell parameters. Larger alkali metals on position A2 and A4 result in an increased value for the a-axis, whereas smaller alkali metals on A1 and A3 result in decreasing values for c. This compression along c upon an increasing content of potassium is also reflected in the decreasing *c/a* value (Table 1 and Table 2).

The values for the quaternary compound in Table 1 and Table 3 represent our best possible model for the structure solution involving three different alkali metals yielding the sum formula Cs_3.57_K_4.55_Rb_6.92_Tl_27_. Of course, a definitive statement about the alkali metal proportions, when three alkali metals are involved, is not possible; therefore, this compound is listed, but will not be discussed in detail.

In general, the size of the pore, which is reflected in the Tl4-A4 and Tl5-A4 distances (Table 3), is not only affected by the size of the (mixed) alkali metals on position A4, but also by the differently occupied remaining alkali metal sites. 

The layer and the cluster separating positions A1 and A3 show fewer contacts within smaller distances, which is equitable to a smaller void; therefore, a preferred occupation by lighter alkali metals should be expected. This can be confirmed by the observed compounds. Again, the occupation tendencies differ for both sites. The alkali metal position next to the two-dimensional Tl_16_^8—^ layer (A3) consistently shows a higher amount of lighter alkali metals than the one next to the Tl_11_ clusters (A1). This is in accordance with the surroundings of A1 and A3, as the distances between the thallium atoms and A3 are smaller (<4.14 Å) than the Tl-A1 distances (d(Tl-A1) < 4.29 Å). In other words, the smallest void is observed around A3, where preferably smaller alkali metal resides.

### 4.2. Influence of Mixed Alkali Metal Sites on the Thallium Substructures

**Figure 2:**
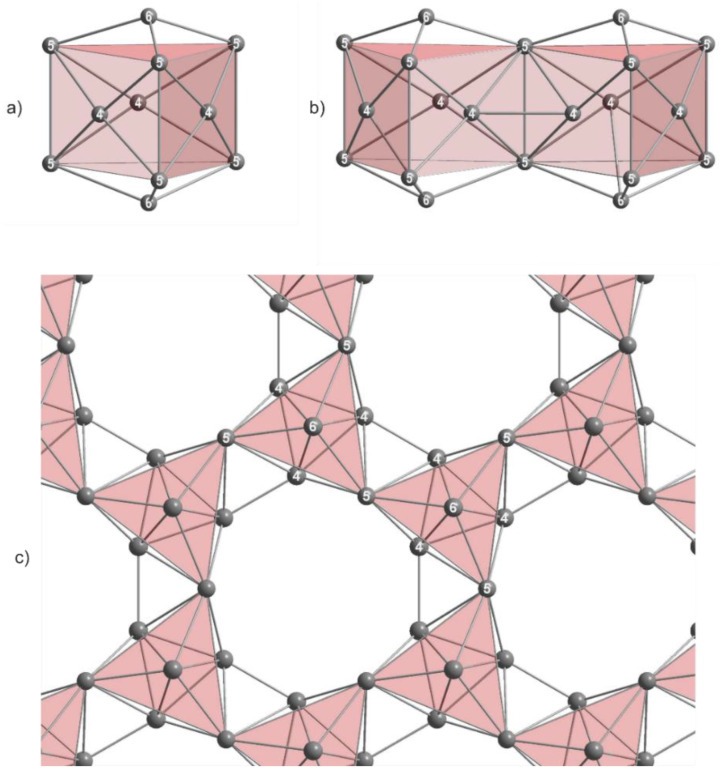
The two-dimensional layer in A_15_Tl_27_ type structures consists of Tl_11_ clusters (**a**) which are interconnected by a common Tl5-Tl5 edge and a Tl4-Tl4 inter-cluster distance is formed (**b**). Altogether, six Tl_11_ cluster entities define the pore (**c**). In A_15_Tl_27_, this pore is filled by alkali metal. Cs_8.21_Rb_6.76_Tl_27.09_ and Cs_14.53_Tl_28.4_ prove the possibility of substituting this alkali metal position by thallium. Selected distances for both compounds are given in Table 5.

In A_15_Tl_27_, isolated Tl_11_ clusters are present; additionally, the two-dimensional layer which can be regarded as connected Tl_11_ clusters via common Tl5-Tl5 edges (Figure 2) is also present.

**Figure 3:**
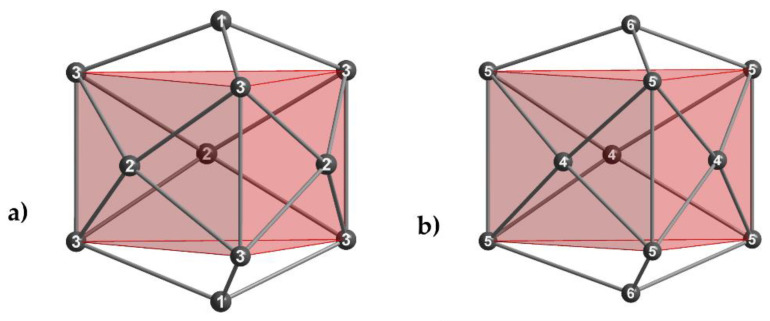
(**a**) isolated Tl_11_ cluster and (**b**) Tl_11_ cluster of the two-dimensional layer.

By comparing those two different Tl_11_ entities, the Tl3-Tl3 distances (isolated Tl_11_^7—^) and Tl5-Tl5 distances (Tl_16_^8—^ layer) refer to the stretching or compression of the clusters in horizontal and vertical directions (Figure 3a,b).

For the isolated Tl_11_ clusters (Figure 3a, Table 4), the distance from the trigonal prism (Tl3) to the quadrangular face capping Tl2 atoms increases, whereas the distance between the Tl3 atoms diminishes when the potassium proportion is enlarged. This is according to the observed *c/a* value for the unit cell parameters. Generally, Tl3-Tl3 distances in the isolated clusters are shorter compared with the Tl5-Tl5 distances of the fused clusters within the layer, which already was observed by *Corbett* in his three compounds (Rb_15_Tl_27_, Cs_15_Tl_27_ and Rb_14_CsTl_27_) [14]. This trend can be confirmed for our ternary and quaternary thallides.

Ideal Tl_11_^7—^ clusters exhibit D_3*h*_ symmetry. In contrast, the Tl_11_ cluster fragment of the two-dimensional layer shows C_3*h*_ symmetry due to missing vertical mirror planes for this point group. As a result, two different values for Tl4-Tl5 distances are observed. For the evaluation of the distortion of the layer, the deviation from C*_3h_* to the higher D_3*h*_ symmetry can be taken into account. For this purpose, we already introduced a *cdd*/*cd_av_* ratio (*cdd*: capping distance difference; *cd_av_*: average capping distance; Equation (1)) for isolated Tl_11_^7—^ clusters [18], which allows a quick estimation of the degree of distortion. This approach was employed again in order to gain deeper insight in the degree of distortion of the layer-forming Tl_11_ entities.

(1) $$\frac{cdd}{cd_{av}}=\frac{\left|cd_{2}-cd_{1}\right|}{\left(\frac{cd_{2}+cd_{1}}{2}\right)}\;\;\;with\;cd_{1}\leq \;cd_{2}$$

It becomes apparent, that as soon as potassium is present, the degree of distortion gives larger values compared with compounds without potassium. The K-Rb approach shows the greatest degree of distortion with almost 7%. This indicates that additional distortion can be expected when rubidium is used instead of cesium, which we focused on in our current investigations. Considering the Cs-K approaches, the degree of distortion increases with increasing potassium content (Table 5). Therefore, the substitution of cesium by lighter alkali metals has a clear influence on the two-dimensional Tl layer structure.

### 4.3. Effects of Incorporation of Tl in the Two-Dimensional Layers

**Figure 4:**
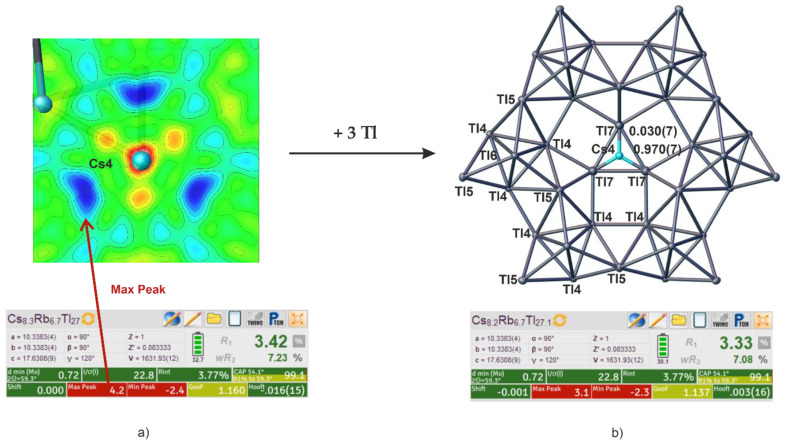
(**a**) Electron density map generated by Olex2 (see Section 2) of the area around Cs4 shows surrounding residual density (blue), which created the idea of additional thallium being partially present instead of Cs4. (**b**) The partial replacement of Cs4 (Wyckoff 1b) by Tl7 (Wyckoff 3g) results in two subunits being present in Cs_8.21_Rb_6.76_Tl_27.1_: cesium, including Tl_16_^8−^ layers and cesium-free Tl_19_^7−^ layers. Refinement indicators (Olex2) show improved model for Cs_8.21_Rb_6.76_Tl_27.1_.

The large pores in the two-dimensional layer were shown to be preferably occupied by larger alkali metals. In K_14_Cd_9_Tl_21_ [22], it was reported, that instead of alkali metal also cadmium can be present, yielding an alkali metal-free [Cd_9_Tl_10_]^7—^ layer. Unusually large residual electron density beside cesium in cadmium-free Cs_8.22_Rb_6.75_Tl_27.09_ created the idea that it might be possible to introduce thallium in this place. In this compound, the electron density was refined to a value of 3% for thallium (see Figure 4), which of course does not prove this theory. 

**Figure 5:**
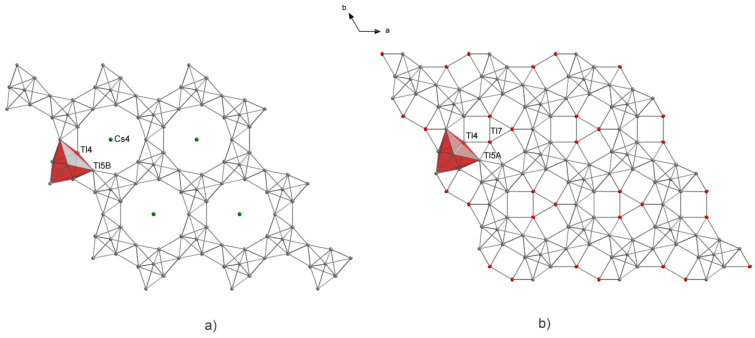
Different hosts in the pores of the two-dimensional layer of A_15_Tl_27_-type structures: (**a**) Cs_15_Tl_27_; (**b**) Cs_14.53_Tl_28.4_.

**Figure 6:**
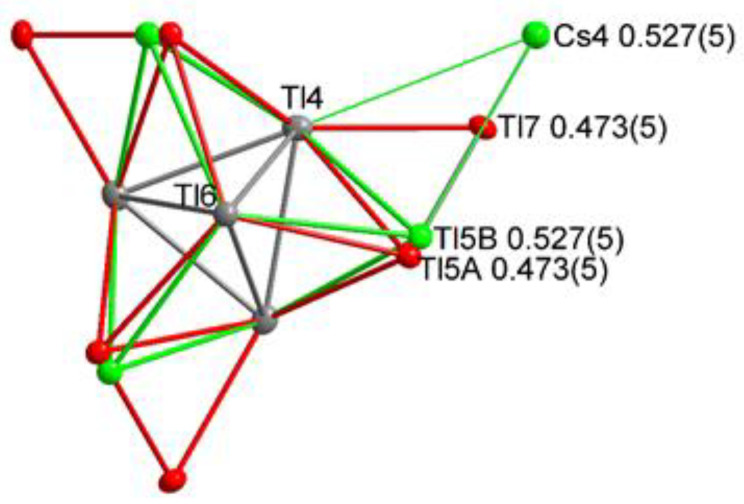
When Cs4 is replaced by Tl7, the unusually short Tl7-Tl5 distance would be observed. Prolate displacement of Tl5 indicated split positions, which were refined according to the s.o.f. of Tl7 and Cs4, respectively. This model yields a reasonable Tl5A-Tl7 distance and improved residual density description.

Subsequently, larger amounts of thallium were employed during the solid-state synthesis to prove the idea of thallium being embedded into the pores of the Tl_16_^8—^ layers of Cs_15_Tl_27_ (see Section 3. Results). The obtained single crystals undoubtedly confirmed the presence of additional thallium in the pores to an extent of 0.473(5). The s.o.f.’s of Cs4 accordingly reduced to 0.527(5)). Additionally, split positions for Tl5 could be refined, which are induced by the thallium incorporation in the pore (Figure 5 and Figure 6). This leads to two settings being present in Cs_14.53_Tl_28.4_: On the one hand a pore description is obtained, equivalent to Cs_15_Tl_27_, with two-dimensional [Tl_16_]^8—^ layers with cesium (Cs4, 1*b*) residing in the pore (Figure 5a). On the other hand, pores are present, where instead of cesium on the Wyckoff position 1*b*, three thallium atoms (Tl7, 3*g*) are present in the [Tl_19_]^7—^ layer (Figure 5b). The assignment of the charge of the layer is due to the known charge of the Tl_11_^7—^ clusters [14] and by assuming a complete electron transfer from the alkali metal to thallium, which is according to the approach of *Tillard* et al. and *Corbett* et al. In comparison with the cadmium compound, where Cd2 atoms form a triangle in the pores, the distances between the Tl7 atoms in our triangle are longer (d(Cd2-Cd2) = 2.816(7) Å [22], d(Tl7-Tl7) = 3.126(4) Å). The two symmetrically inequivalent Tl4-Tl4 distances in Cs_14.53_Tl_28.4_ (3.2860(14) Å and 3.1423(15) Å) become more similar compared with Tl4-Tl4 in Cs_15_Tl_27_ (3.322(2) Å and 3.102(3) Å). In the related compound K_14_Cd_9_Tl_21_, a similar trend is noticed [22].

The change in the overall thallium substructure of the layer upon thallium substitution can be directly demonstrated by the position of Tl5. This position showed prolate anisotropic displacement, which could be reduced by introducing split positions. The free refinement of the split Tl positions gives s.o.f. values of 0.527(16) and 0.473(16). As these values are according to the s.o.f.s of Cs4 (0.527(5)) and Tl7 (0.473(5)), respectively, the free refinement of the split position was performed using the same s.o.f. parameter. The reason for this movement of Tl5 upon Tl substitution can be found in the newly formed Tl7-Tl5 distance: if Tl5 was not split, this would mean a short Tl5-Tl7 distance (<2.900(2) Å), which seems to be unfavorable. The splitting of this position demonstrates how the layer structure is able to respond to a change of the host in the pores. As in the above-discussed A_15_Tl_27_ compounds, a degree of distortion within the layer can be calculated. In the present case of Cs_14.53_Tl_28.4_, two different degrees of distortion are observed, as the Tl5 position is split (see Figure 5 and Figure 6). The partial structure with the additionally embedded thallium shows a significantly smaller degree of distortion compared with all other compounds (4.3%). The second partial structure with A4 occupied by Cs gives a degree of distortion similar to that of the Cs-K phases (see Table 5). 

Altogether, the formal oxidation of the former Tl_16_^8—^ layer by forming layers of Tl_19_^7—^ upon thallium substitution yields a less distorted thallium substructure. We observed similar effects previously for A_8_Tl_11_X, where less distorted Tl_11_^7—^ was observed when halide was incorporated [18], which is equivalent to a formal oxidation of thallium in A_8_Tl_11_. A speculative compound A_14_Tl_30_ would mean that solely cesium-free layers are present. Further attempts to increase the thallium content by using the mixed alkali metal approach are currently in progress.

## 5. Conclusions

In summary, it can be stated that substitution of the larger alkali metals in the A_15_Tl_27_ type structure by potassium is possible to a certain extent. The presence of large alkali metals in the pores of the two-dimensional Tl_16_^8—^ layer is essential for the stabilization of the A_15_Tl_27_ type structure. If the amount of potassium is enlarged, instead of binary K_15_Tl_27_, only K_49_Tl_108_ is observed, which is the more stable phase at appr. 1:2 (A:Tl) composition involving this lighter homologue of the alkali metals. Therefore, Cs_2.27_K_12.73_Tl_27_ is currently the first and at the same time the potassium-richest compound found in the A_15_Tl_27_ type structure. The change in alkali metals is also reflected in the distortion of the Tl_16_^8—^ layer structure. When cesium is involved, less distorted layer structures are observed. 

Furthermore, it could be shown in Cs_14.53_Tl_28.4_, that cesium in the pore of Cs_15_Tl_27_ can be partially substituted by three thallium atoms yielding formally oxidized, less distorted two-dimensional Tl_19_^7—^ layers.

## Figures and Tables

**Table 1:** Crystal data and structure refinement details of the approaches to obtain binary K_15_Tl_27_.

Empirical Formula	K_6.96_Rb_8.04_Tl_27_	Cs_5.92_K_9.08_Tl_27_	Cs_2.27_K_12.73_Tl_27_	Cs_3.57_K_4.55_Rb_6.92_Tl_27_
CSD number *	2088508	2093385	2093386	2093391
Formula weight	6477.30	6659.84	6318.00	6761.84
Temperature (K)	123	123	123	123
Crystal system	hexagonal	hexagonal	hexagonal	hexagonal
Space group	*P*-62*m*	*P*-62*m*	*P*-62*m*	*P*-62*m*
a (Å)	10.1835(2)	10.2542(4)	10.20330(10)	10.30543(11)
c (Å)	17.1041(4)	17.0278(12)	16.7702(2)	17.2475(2)
α (°)	90	90	90	90
γ (°)	120	120	120	120
Volume (Å^3^)	1536.12(7)	1550.57(17)	1511.99(3)	1586.31(4)
*c/a*	1.68	1.66	1.64	1.67
Z	1	1	1	1
ρ_calc_ (g/cm^3)^	7.002	7.132	6.938	7.078
µ (mm^−1^)	77.292	73.869	73.847	75.853
F(000)	2617.0	2685.0	2554.0	2726.0
Crystal size (mm^3^)	0.12 × 0.09 × 0.08	0.183 × 0.113 × 0.044	0.093 × 0.058 × 0.04	0.105 × 0.051 × 0.039
Radiation	MoKα	MoKα	MoKα	MoKα
	(λ = 0.71073)	(λ = 0.71073)	(λ = 0.71073)	(λ = 0.71073)
2Θ range for data collection (°)	6.636 to 59.01	6.63 to 59.508	6.698 to 70.162	6.57 to 72.68
Index ranges	−10 ≤ h ≤ 12	−13 ≤ h ≤ 14	−16 ≤ h ≤ 16	−16 ≤ h ≤ 17
	−13 ≤ k ≤ 5	−11 ≤ k ≤ 12	−16 ≤ k ≤ 16	−17 ≤ k ≤ 17
	−11 ≤ l ≤ 23	−23 ≤ l ≤ 23	−27 ≤ l ≤ 26	−28 ≤ l ≤ 28
Reflections collected	3380	7018	97696	103808
Independent reflections	1431	1526	2479	2828
Data/restraints/parameters	1431/0/48	1526/0/47	2479/0/47	2828/3/52
Goodness-of-fit on F^2^	1.079	1.111	1.359	1.235
R_int_	R_int_ = 0.0352	R_int_ = 0.0660	R_int_ = 0.0481	R_int_ = 0.0611
Final R indexes	R1 = 0.0419	R1 = 0.0345	R1 = 0.0173	R1 = 0.0208
[I >= 2σ (I)]	wR2 = 0.1003	wR2 = 0.0631	wR2 = 0.0504	wR2 = 0.0592
Final R indexes	R1 = 0.0442	R1 = 0.0404	R1 = 0.0185	R1 = 0.0221
[all data]	wR2 = 0.1031	wR2 = 0.0655	wR2 = 0.0506	wR2 = 0.0596
Largest diff. peak/hole (e Å^−3^)	2.04/−1.94	2.59/−2.62	3.37/−2.07	2.78/−2.06
Flack parameter	0.03(7)	−0.006(16)	0.005(4)	0.003(5)

* Further details of the crystal structure investigation(s) may be obtained free of charge from the Cambridge Crystallographic Data Centre CCDC (Access Structures) on quoting the deposition number (given in the table) CSD-xxxxxx or the the deposition number CCDC-xxxxxxx.

**Table 2:** Crystal data and structure refinement details of the approaches with incorporated Tl.

Empirical Formula	Cs_8.21_Rb_6.76_Tl_27.09_	Cs_14.53_Tl_28.4_	Cs_15_Tl_27_
CSD number *	2088490	2088509	2088513
Formula weight	7205.35	7735.60	7511.64
Temperature (K)	122.99(10)	123.00(16)	123.00(18)
Crystal system	hexagonal	hexagonal	hexagonal
Space group	*P*-62*m*	*P*-62*m*	*P*-62*m*
a (Å)	10.3383(4)	10.5007(3)	10.4240(7)
c (Å)	17.6308(9)	17.9963(6)	18.0525(16)
α (°)	90	90	90
γ (°)	120	120	120
Volume (Å^3^)	1631.93(15)	1718.50(11)	1698.8(3)
*c/a*	1.71	1.71	1.73
Z	1	1	2
ρ_calc_ (g/cm^3^)	7.332	7.474	14.685
μ (mm^−1^)	76.096	73.862	143.327
F(000)	2896.0	3100.0	6024.0
Crystal size (mm^3^)	0.06 × 0.05 × 0.04	0.051 × 0.045 × 0.036	0.05 × 0.032 × 0.016
Radiation	MoKα	MoKα	MoKα
	(λ = 0.71073)	(λ = 0.71073)	(λ = 0.71073)
2Θ range for data collection (°)	6.486 to 59.35	7.762 to 66.274	7.82 to 56.438
Index ranges	−13 ≤ h ≤ 13,	−16 ≤ h ≤ 15,	−11 ≤ h ≤ 12,
	−13 ≤ k ≤ 13,	−16 ≤ k ≤ 16,	−13 ≤ k ≤ 13,
	−24 ≤ l ≤ 14	−27 ≤ l ≤ 26	−21 ≤ l ≤ 24
Reflections collected	4416	14514	8330
Independent reflections	1536	2408	1570
Data/restraints/parameters	1536/0/50	2408/6/56	1570/0/45
Goodness-of-fit on F^2^	1.178	1.062	1.069
R_int_	R_int_ = 0.0377	R_int_ = 0.0473	R_int_ = 0.0881
Final R indexes [I >= 2σ (I)]	R1 = 0.0333	R1 = 0.0281	R1 = 0.0376
	wR2 = 0.0656	wR2 = 0.0591	wR2 = 0.0634
Final R indexes [all data]	R1 = 0.0384	R1 = 0.0325	R1 = 0.0495
	wR2 = 0.0675	wR2 = 0.0606	wR2 = 0.0671
Largest diff. peak/hole (e Å^−3^)	3.12/−2.33	1.48/−1.51	2.42/−2.30
Flack parameter	0.002(16)	−0.004(9)	−0.017(19)

* Further details of the crystal structure investigation(s) may be obtained free of charge from the Cambridge Crystallographic Data Centre CCDC (Access Structures) on quoting the deposition number (given in the table) CSD-xxxxxx or the the deposition number CCDC-xxxxxxx.

**Table 3:** Site occupancy factors (s.o.f.) of the symmetry-independent alkali metal positions of the mixed alkali metal thallides of the A_15_Tl_27_ type structure.

Compound	A1 (6*i*)	A2 (2*c*)	A3 (6*i*)	A4 (1*b*)	d(Tl4-A4) (Å) d(Tl5-A4) (Å)
Cs_8.21_Rb_6.76_Tl_27.09_	Cs 0.57(2)	Cs	Cs 0.31(2)	Cs 0.970(7)	4.2212(11)
	Rb 0.43(2)		Rb 0.69(2)	Tl 0.030(7)	4.2995(12)
K_6.96_Rb_8.04_Tl_27_	K 0.45(3)	Rb	K 0.68(3)	K 0.18(6)	4.1369(13)
	Rb 0.55(3)		Rb 0.32(3)	Rb 0.82(6)	4.2369(15)
Cs_5.85_K_9.15_Tl_27_	Cs 0.368(10)	Cs	Cs 0.119(9)	Cs	4.1786(9)
	K 0.632(10)		K 0.881(9)		4.2623(11)
Cs_2.27_K_12.73_Tl_27_	K	Cs 0.665(13)	K	Cs	4.1475(5)
		K 0.335(13)			4.2584(5)
Cs_3.57_K_4.55_Rb_6.92_Tl_27_	Cs 0.18(3) Rb 0.56(5) K 0.26(3)	Cs 0.75(3) Rb 0.25(4)	Rb 0.508(16) K 0.49(3)	Cs	4.1996(5) 4.2885(6)

**Table 4:** Dimensions of the isolated Tl_11_-clusters in A_15_Tl_27_ structure type.

Compound	d(Tl2-Tl2) (Å)	d(Tl3-Tl3) (Å)	d(Tl2-Tl3) (Å)
Cs_8.21_Rb_6.76_Tl_27.09_	3.749(3)	3.202(2)	3.0710(10)
K_6.96_Rb_8.04_Tl_27_	3.753(3)	3.198(2)	3.0855(11)
Cs_5.85_K_9.15_Tl_27_	3.721(2)	3.197(2)	3.0731(9)
Cs_2.27_K_12.73_Tl_27_	3.7333(12)	3.1908(10)	3.0926(4)
Cs_14.53_Tl_28.4_	3.7697(16)	3.2146(13)	3.0833(6)
Cs_15_Tl_27_	3.774(3)	3.212(3)	3.0816(12)

**Table 5:** Dimensions of the Tl_11_ clusters in the two-dimensional layers of the A_15_Tl_27_ type structure. The atom numbers are according to the numbering scheme in Figure 4. A and B indicate the split positions of Tl5.

Compound	d(Tl6-Tl5) (Å)	d(Tl5-Tl5) (Å)	d(Tl4-Tl4) (Å)	d(Tl4-Tl5) (Å)	d(Tl4-Tl5#) (Å)	d(Tl4-Tl6) (Å)	$\frac{cdd}{cd_{av}}(\%)$	*c/a*
Cs_8.21_Rb_6.76_Tl_27.09_	3.3735(7)	3.463(2)	3.2924(19) 3.088(2)	3.2122(11)	3.3917(14)	3.3078(13)	5.44	1.71
K_6.96_Rb_8.04_Tl_27_	3.3423(8)	3.533(2)	3.270(2) 3.040(3)	3.1796(13)	3.4068(17)	3.3450(15)	6.90	1.68
Cs_5.85_K_9.15_Tl_27_	3.3622(7)	3.475(2)	3.2721(16) 3.0713(19)	3.1860(10)	3.3923(12)	3.3329(13)	6.27	1.66
Cs_2.27_K_12.73_Tl_27_	3.3493(3)	3.5211(9)	3.2665(8) 3.0606(9)	3.1798(5)	3.3981(6)	3.3611(5)	6.64	1.64
Cs_14.53_Tl_28.4_	3.3093(15) (A) 3.4628(19) (B)	3.627(10) (A) 3.457(11) (B)	3.2860(14) 3.1423(15)	3.242(4) (A) 3.259(3) (B)	3.385(3) (A) 3.465(4) (B)	3.3170(8)	4.32 6.13	1.71
Cs_15_Tl_27_	3.3940(8)	3.457(3)	3.322(2) 3.102(3)	3.2361(14)	3.4039(17)	3.3026(16)	5.05	1.73

## Data Availability

Further details on the crystal structure investigation(s) may be obtained free of charge from The Cambridge Crystallographic data center CCDC (Access Structures) on quoting the deposition number given in the crystallographic tables (CSD-xxxxxx or the deposition number CCDC-xxxxxxx).

## Supplementary Materials

The following are available online at https://www.mdpi.com/article/10.3390/ma14247512/s1, Figure S1: Measured powder diffraction patterns of Cs15Tl27 (pink) and Cs14.53Tl28.4 (blue); Figure S2: Measured powder diffraction pattern of Cs 2.27 K 12.73 Tl 27 (blue) with calculated reflections of K 49 Tl 108 (pink) and of Cs 2.27 K 12.73 Tl 27from single crystal data; Figure S3: Measured powder diffraction patter of Cs 2.27 K 12.73 Tl 27 (blue, up) with calculated reflections of K 49 Tl 108 (purple, up)and calculated pattern of Cs 2.27 K 12.73 Tl 27 from single crystal (blue, down)(*) unindexed lines, impurities
