# Supplementary material for: The Role of Different Alkali Metals in the A15Tl27 Type Structure and the Synthesis and X-ray Structure Analysis of a New Substitutional Variant Cs14.53Tl28.4
Source: Materials (Basel). 2021 Dec 8;14(24):7512. doi: 10.3390/ma14247512 (PMC8705008; doi:10.3390/ma14247512)
Supplement: Supplementary file 1 [file materials-14-07512-s001.zip › materials-1463749-supplementary.pdf]

The role of different alkali metals in the $A_{15}Tl_{27}$ type structure and the synthesis and X-Ray structure analysis of a new substitutional variant $Cs_{14.53}Tl_{28.4}$

Supplementary Material

Vanessa F. Schwinghammer[1], Susanne M. Tiefenthaler[1], Stefanie Gärtner[1,2*]

[1]Institute of Inorganic Chemistry, University of Regensburg, 93040 Regensburg, Germany, Vanessa.Schwinghammer@ur.de, Susanne.Tiefenthaler@ur.de

[2]Central Analytics, X-Ray Crystallography Dept., University of Regensburg, 93040 Regensburg, Germany, Stefanie.Gaertner@ur.de

[*]Correspondence: Stefanie.Gaertner@ur.de, Tel.: +49-941-943-4446

1. PXRD Patterns

1.1 Powder diffraction pattern of $Cs_{15}Tl_{27}$ and $Cs_{14.53}Tl_{28.4}$

1.2 Powder diffraction pattern of $Cs_{2.27}K_{12.73}Tl_{27}$ compact

1.3 Powder diffraction pattern of $Cs_{2.27}K_{12.73}Tl_{27}$

2. Indexing of PXRD patterns

2.1 $Cs_{15}Tl_{27}$

2.2 $Cs_{14.53}Tl_{28.4}$

2.2 $Cs_{2.27}K_{12.73}Tl_{27}$

1.1 Powder diffraction pattern of $Cs_{15}Tl_{27}$ and $Cs_{14.53}Tl_{28.4}$

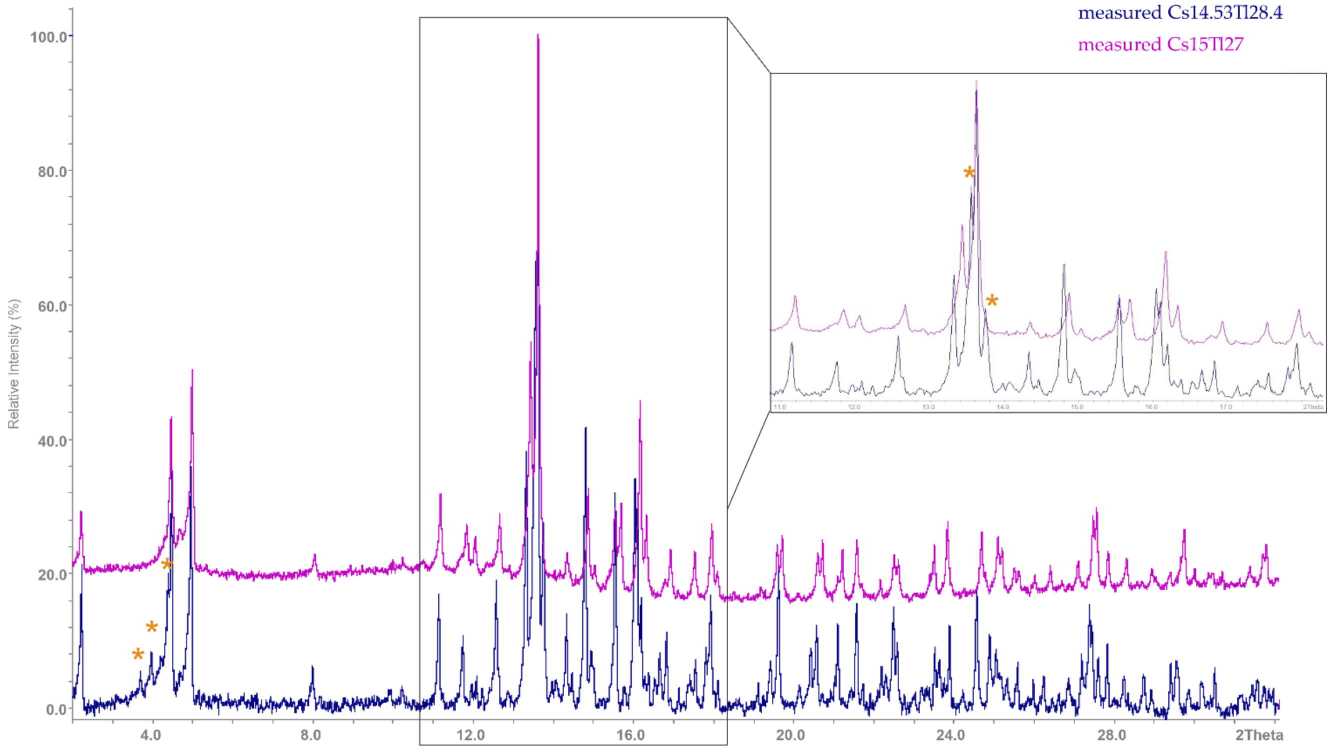

Figure S1: Measured powder diffraction patterns of $Cs_{15}Tl_{27}$ (pink) and $Cs_{14.53}Tl_{28.4}$ (blue)
(*) unindexed lines, impurities

1.2 Powder diffraction pattern of $Cs_{2.27}K_{12.73}Tl_{27}$ compact

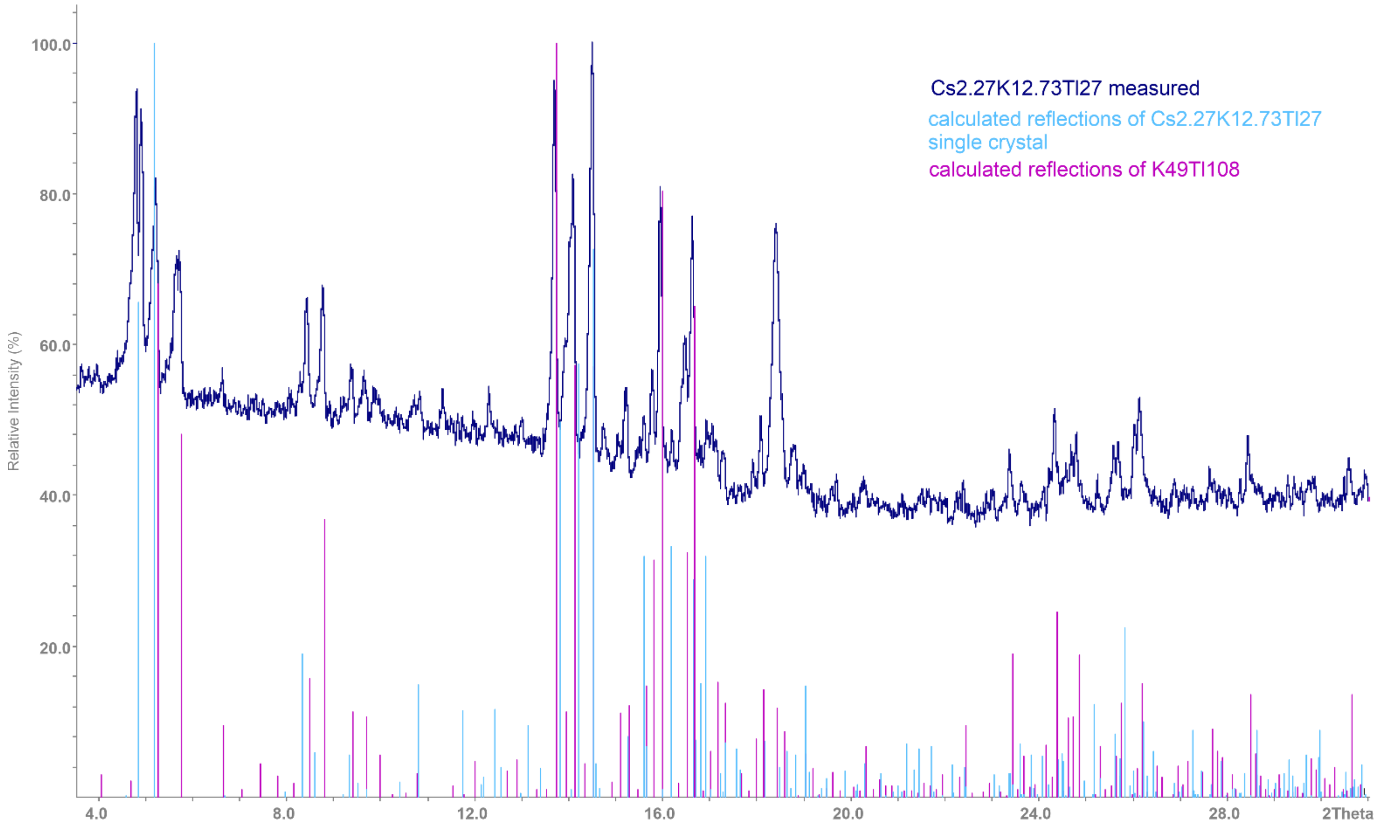

Figure S2: Measured powder diffraction pattern of $Cs_{2.27}K_{12.73}Tl_{27}$ (blue) with calculated reflections of $K_{49}Tl_{108}$ (pink) and of $Cs_{2.27}K_{12.73}Tl_{27}$ from single crystal data

1.3 Powder diffraction pattern of $Cs_{2.27}K_{12.73}Tl_{27}$

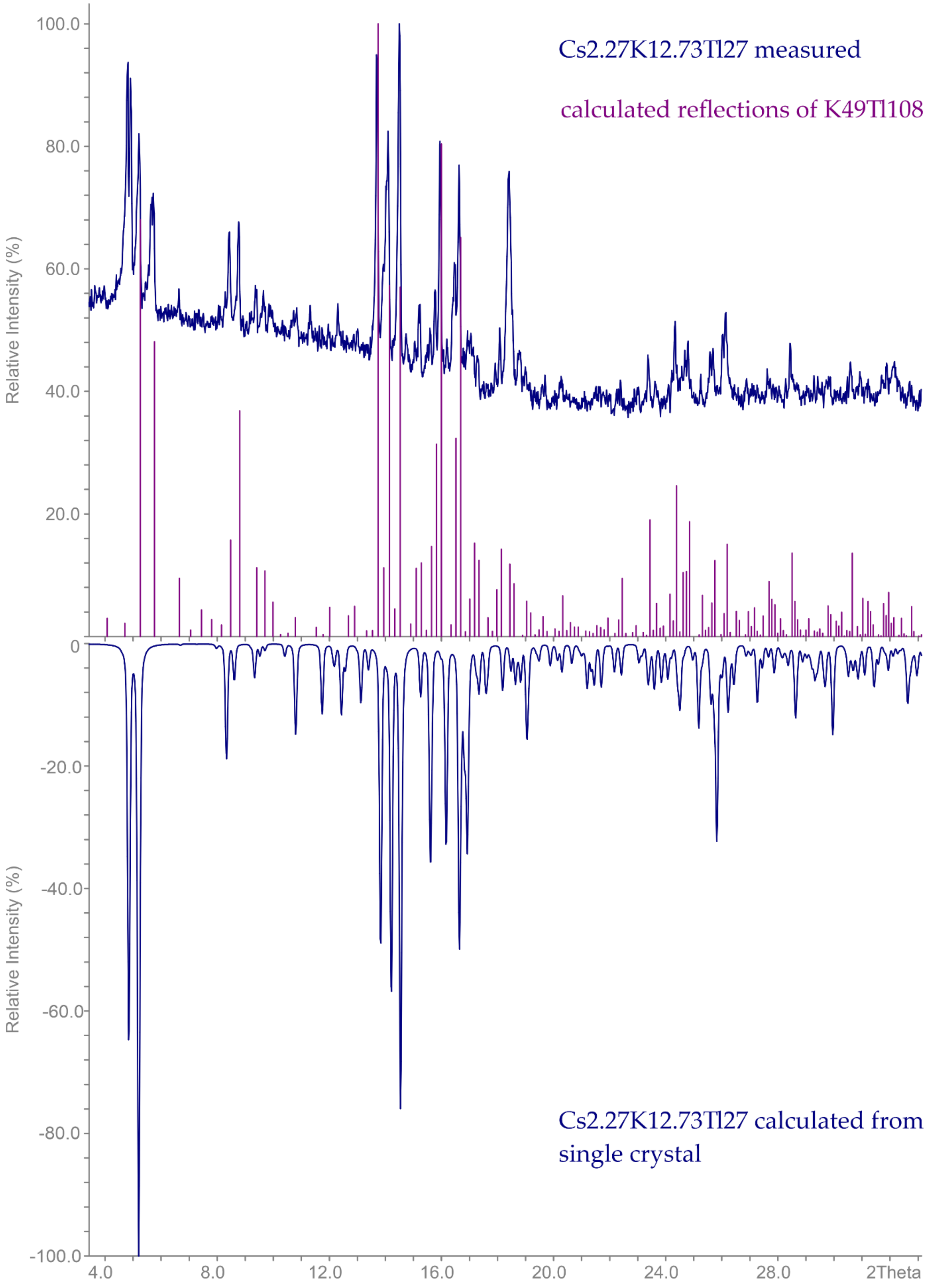

Figure S3: Measured powder diffraction patter of $Cs_{2.27}K_{12.73}Tl_{27}$ (blue, up) with calculated reflections of $K_{49}Tl_{108}$ (purple, up) and calculated pattern of $Cs_{2.27}K_{12.73}Tl_{27}$ from single crystal (blue, down)

2. Indexing PXRD patterns

2.1 $Cs_{15}Tl_{27}$

Wavelength : 0.709300 Å
Symmetry : Hexagonal P
Spacegroup : P -6 2 m
Refined cell parameters:
a = 10.501(5) Å
c = 18.157(5) Å
V = 1734.1(10) Å^3
Number of single indexed lines : 17
Number of unindexed lines : 0

N	2Th[obs]	H	K	L	2Th[calc]	obs-calc	Int.	d[obs]	d[calc]
1	2.217	0	0	1	2.238	-0.0219	29.7	183.363	181.573
2	4.459	1	0	0	4.470	-0.0111	44.3	91.172	90.946
		0	0	2	4.478	-0.019			90.786
3	4.983	1	0	1	4.999	-0.0163	51.1	81.581	81.316
4	10.008	2	0	2	10.008	-0.0004	23.3	40.659	40.658
		1	0	4	10.019	-0.011			40.615
5	10.239	1	1	3	10.260	-0.0209	23.5	39.743	39.662
6	11.181	2	0	3	11.196	-0.0153	32.3	36.405	36.355
		0	0	5	11.209	-0.0279			36.315
7	11.825	2	1	0	11.844	-0.0185	26.1	34.428	34.374
8	12.045	2	1	1	12.055	-0.0105	26	33.804	33.774
		1	0	5	12.073	-0.0281			33.725
9	12.653	2	1	2	12.668	-0.0149	27.9	32.185	32.147
		2	0	4	12.676	-0.0233			32.126
10	13.432	3	0	0	13.437	-0.0049	56.1	30.326	30.315
		0	0	6	13.460	-0.0285			30.262
11	13.624	3	0	1	13.623	0.0004	100	29.900	29.901
		2	1	3	13.629	-0.0048			29.890
		1	1	5	13.639	-0.0152			29.867
12	14.346	2	0	5	14.359	-0.0129	23.2	28.402	28.376
13	14.865	2	1	4	14.872	-0.007	32.4	27.417	27.404
14	15.030	3	0	3	15.036	-0.0064	22.3	27.117	27.105
15	15.528	2	2	0	15.527	0.0006	27.8	26.253	26.254
		1	1	6	15.548	-0.02			26.219
16	15.701	2	2	1	15.690	0.0117	32.1	25.964	25.984
		0	0	7	15.717	-0.0154			25.939
17	16.163	3	1	0	16.165	-0.0023	47.8	25.227	25.224
		2	2	2	16.168	-0.0045			25.220
		3	0	4	16.174	-0.0111			25.210
		2	0	6	16.185	-0.0221			25.193

18	16.326	3	1	1	16.322	0.0046	30.3	24.977	24.984
		2	1	5	16.335	-0.0084			24.964
		1	0	7	16.348	-0.0215			24.944
19	16.766	3	1	2	16.782	-0.0161	19.4	24.326	24.303
20	16.924	2	2	3	16.935	-0.0103	23.6	24.100	24.085
21	17.522	3	1	3	17.523	-0.0009	22.7	23.284	23.283
		3	0	5	17.531	-0.0091			23.272
		1	1	7	17.543	-0.0212			23.256
22	17.951	4	0	0	17.948	0.0029	27.1	22.733	22.736
		2	2	4	17.956	-0.005			22.726
		2	1	6	17.966	-0.0149			22.714
		0	0	8	17.979	-0.0288			22.697
23	18.085	4	0	1	18.089	-0.0038	20.9	22.565	22.560
		2	0	7	18.113	-0.0274			22.531
24	19.195	4	0	3	19.183	0.0115	18.2	21.271	21.284
		2	2	5	19.191	0.0041			21.276
25	19.597	3	2	0	19.573	0.0242	24.2	20.839	20.864
		1	1	8	19.602	-0.005			20.834
26	19.702	3	2	1	19.703	-0.0014	24.8	20.729	20.728
		3	1	5	19.714	-0.0122			20.717
		2	1	7	19.725	-0.0231			20.705
27	20.098	3	2	2	20.089	0.0095	17.7	20.325	20.334
		4	0	4	20.094	0.0042			20.329
		2	0	8	20.115	-0.0171			20.308
28	20.596	4	1	0	20.588	0.008	23.1	19.838	19.846
		2	2	6	20.604	-0.0076			19.831
29	20.712	4	1	1	20.712	-0.0006	24.5	19.729	19.728
		3	2	3	20.716	-0.0041			19.725
		3	0	7	20.733	-0.0214			19.709
30	21.105	4	1	2	21.080	0.0256	20.7	19.365	19.388
		3	1	6	21.094	0.012			19.376
31	21.204	4	0	5	21.210	-0.0056	22.6	19.276	19.271
32	21.573	3	2	4	21.564	0.0088	24.6	18.950	18.958
		2	1	8	21.584	-0.0112			18.940
33	22.161	2	2	7	22.163	-0.0021	18.7	18.454	18.452
		2	0	9	22.176	-0.015			18.441
34	22.509	5	0	0	22.487	0.0222	23.3	18.171	18.189
		4	1	4	22.493	0.0158			18.184
		4	0	6	22.501	0.0078			18.178
		3	0	8	22.513	-0.0034			18.169
		0	0	10	22.527	-0.0177			18.157
35	22.610	5	0	1	22.601	0.0088	22.2	18.092	18.099
		3	2	5	22.611	-0.0008			18.091
		3	1	7	22.620	-0.0103			18.083
36	23.397	3	3	0	23.381	0.0154	20.7	17.491	17.502
37	23.502	3	3	1	23.491	0.0104	23.8	17.414	17.422

		5	0	3	23.494	0.0073			17.419
		4	1	5	23.501	0.0012			17.415
		2	1	9	23.522	-0.0203			17.399
38	23.837	4	2	0	23.817	0.0205	27.9	17.173	17.187
		3	3	2	23.818	0.019			17.186
		3	2	6	23.830	0.0069			17.177
		2	2	8	23.841	-0.0037			17.170
		1	1	10	23.855	-0.0173			17.160
39	24.693	4	1	6	24.679	0.0141	27.6	16.586	16.596
40	24.797	4	2	3	24.773	0.024	21.3	16.518	16.533
		0	0	11	24.814	-0.0168			16.507
41	25.106	5	1	0	25.080	0.0258	25.9	16.318	16.334
		3	3	4	25.086	0.02			16.331
42	25.202	5	1	1	25.183	0.0192	25	16.256	16.269
		5	0	5	25.191	0.0106			16.263
		3	2	7	25.200	0.002			16.258
		1	0	11	25.226	-0.0238			16.241
43	25.503	5	1	2	25.489	0.0137	21.6	16.068	16.076
		4	2	4	25.494	0.0094			16.074
		4	0	8	25.511	-0.0076			16.063
		2	1	10	25.523	-0.0204			16.055
44	25.619	2	2	9	25.618	0.0011	21.4	15.996	15.997
45	26.009	5	1	3	25.993	0.0166	19.8	15.760	15.770
		3	3	5	25.998	0.011			15.767
		4	1	7	26.006	0.0026			15.762
		3	1	9	26.018	-0.0085			15.755
		1	1	11	26.032	-0.0225			15.747
46	26.402	4	2	5	26.393	0.0094	20.3	15.530	15.535
		2	0	11	26.426	-0.0236			15.516
47	26.694	5	1	4	26.682	0.0121	19.5	15.363	15.370
		3	2	8	26.699	-0.0043			15.360
48	27.106	0	0	12	27.111	-0.0045	21.2	15.134	15.131
49	27.477	4	2	6	27.455	0.0223	29.3	14.933	14.945
		4	1	8	27.464	0.013			14.940
		2	2	10	27.476	0.0011			14.934
		1	0	12	27.491	-0.0135			14.926
50	27.546	4	3	1	27.538	0.0085	29.4	14.896	14.901
		5	1	5	27.546	0.0006			14.897
		5	0	7	27.554	-0.0073			14.893
51	27.844	4	3	2	27.820	0.0242	23.3	14.740	14.753
		3	1	10	27.851	-0.0072			14.736
52	28.294	5	2	1	28.282	0.0113	21.3	14.511	14.516
		4	3	3	28.285	0.0087			14.515
		3	3	7	28.298	-0.0042			14.509
		3	2	9	28.308	-0.0145			14.503
		3	0	11	28.321	-0.0274			14.497

53	28.940	4	3	4	28.924	0.0158	21.4	14.193	14.201
		5	0	8	28.939	0.0006			14.194
		4	0	10	28.951	-0.0107			14.188
54	29.391	6	0	5	29.374	0.0171	20.8	13.980	13.988
		2	2	11	29.404	-0.0128			13.974
		0	0	13	29.419	-0.0278			13.967
55	29.657	6	1	0	29.632	0.0255	23.1	13.857	13.869
		5	2	4	29.637	0.0206			13.867
		3	3	8	29.651	0.0058			13.860
		2	1	12	29.676	-0.0189			13.849
56	29.727	6	1	1	29.720	0.0074	25.2	13.825	13.829
		4	3	5	29.727	0			13.825
		5	1	7	29.735	-0.0074			13.822
		3	1	11	29.757	-0.0295			13.812
57	30.399	3	0	12	30.373	0.0261	20.6	13.527	13.538
		6	1	3	30.418	-0.0192			13.519
		5	2	5	30.423	-0.024			13.517
58	31.396	2	2	12	31.391	0.0052	21.1	13.108	13.110
59	31.702	7	0	0	31.682	0.0194	23.9	12.984	12.992
		4	4	2	31.683	0.0183			12.992
		3	1	12	31.724	-0.0224			12.976
60	31.806	4	3	7	31.779	0.0265	25.2	12.943	12.954
		3	2	11	31.800	0.0056			12.945
		2	1	13	31.814	-0.0083			12.940
61	32.464	5	2	7	32.435	0.029	21.9	12.687	12.699
		5	1	9	32.444	0.0198			12.695
		4	1	11	32.456	0.0084			12.691
		3	0	13	32.469	-0.0052			12.685
62	32.702	6	1	6	32.674	0.0275	22.2	12.598	12.608
		6	0	8	32.682	0.0195			12.605
		3	3	10	32.692	0.0094			12.601
		4	0	12	32.705	-0.0031			12.597
		1	1	14	32.720	-0.0178			12.591
63	33.021	4	3	8	33.003	0.018	21.9	12.479	12.486
		4	2	10	33.014	0.0079			12.482
		2	0	14	33.040	-0.019			12.472
64	33.414	6	2	3	33.386	0.0283	23.1	12.337	12.347
		4	4	5	33.390	0.0239			12.345
		2	2	13	33.430	-0.0161			12.331
65	33.711	7	0	5	33.706	0.0052	21.9	12.231	12.233
		6	1	7	33.712	-0.0014			12.231
		5	0	11	33.732	-0.0213			12.224
66	33.966	6	2	4	33.938	0.028	22.1	12.142	12.152
		5	1	10	33.961	0.0051			12.144
		2	1	14	33.987	-0.0212			12.135
67	34.652	6	2	5	34.636	0.0155	20.9	11.909	11.914

		4	2	11	34.662	-0.0103			11.905
		4	0	13	34.675	-0.0232			11.901
68	34.986	5	2	9	34.957	0.0291	20.8	11.799	11.808
		1	1	15	34.995	-0.0093			11.795
69	35.517	6	0	10	35.491	0.0266	21	11.628	11.636
		5	0	12	35.502	0.015			11.632
		2	2	14	35.516	0.0013			11.628
		5	4	1	35.540	-0.0223			11.621
70	36.385	8	0	0	36.356	0.0298	20.4	11.359	11.368
		6	3	2	36.357	0.0288			11.368
		4	4	8	36.372	0.0133			11.363
		5	2	10	36.381	0.004			11.360
		4	2	12	36.393	-0.0073			11.357
71	36.692	7	0	8	36.665	0.0268	20.1	11.268	11.276
		4	0	14	36.699	-0.007			11.266
		1	0	16	36.714	-0.0223			11.261
72	37.287	5	1	12	37.265	0.0226	21.2	11.094	11.100
		1	1	16	37.293	-0.0057			11.092
		7	2	1	37.301	-0.0133			11.090
		5	4	5	37.307	-0.0193			11.088
73	37.563	6	1	10	37.541	0.0223	21.1	11.015	11.022
		3	2	14	37.565	-0.0018			11.015
		2	0	16	37.580	-0.0169			11.011
74	37.926	5	2	11	37.902	0.0238	21.6	10.914	10.920
		3	3	13	37.914	0.0118			10.917
		3	1	15	37.928	-0.0021			10.913
75	38.697	6	0	12	38.681	0.0161	21	10.704	10.709
76	39.025	7	2	5	38.999	0.0254	21.6	10.618	10.625
		5	4	7	39.005	0.0195			10.623
		6	1	11	39.023	0.002			10.619
		5	1	13	39.034	-0.0096			10.615
77	39.258	5	0	14	39.248	0.0104	22.4	10.557	10.560
78	39.590	3	2	15	39.598	-0.0079	21.8	10.472	10.470
		1	1	17	39.613	-0.0232			10.466
79	39.825	3	3	14	39.795	0.0298	21.8	10.413	10.420
		2	2	16	39.810	0.0154			10.417
		6	4	1	39.817	0.0083			10.415
		8	0	7	39.828	-0.0032			10.412
80	40.080	4	2	14	40.067	0.0129	21.6	10.349	10.353
		3	1	16	40.081	-0.0014			10.349
		5	5	3	40.090	-0.0104			10.347
		7	1	9	40.107	-0.0275			10.343
81	40.661	8	1	5	40.632	0.0286	23.1	10.208	10.215
		7	2	7	40.638	0.0229			10.213
		7	0	11	40.655	0.006			10.209
		4	3	13	40.666	-0.0052			10.206

82	41.227	5	2	13	41.198	0.0293	22.3	10.073	10.080
		5	0	15	41.211	0.0163			10.077
		3	0	17	41.226	0.0015			10.074
83	42.978	6	2	12	42.948	0.0299	23	0.9681	0.9688
		5	2	14	42.959	0.0182			0.9685
		2	1	18	42.988	-0.0105			0.9679
		6	4	7	42.990	-0.0127			0.9679

2.2 $Cs_{14.53}Tl_{28.4}$

Wavelength : 0.709300 Å
Symmetry : Hexagonal P
Spacegroup : P -6 2 m
Refined cell parameters:
a = 10.8580(19) Å
c = 18.108(3) Å
V = 1757.1(4) Å³
Number of single indexed lines : 46
Number of unindexed lines : 0

N	2Th[obs]	H	K	L	2Th[calc]	obs-calc	Int.	d[obs]	d[calc]
1	2.236	0	0	1	2.244	-0.0082	24.7	181.748	181.083
2	4.472	0	0	2	4.490	-0.0172	35.5	90.889	90.542
3	4.951	1	0	1	4.971	-0.0199	39.2	82.115	81.786
4	8.010	1	1	1	8.007	0.0031	46.9	50.780	50.799
5	11.135	2	0	3	11.151	-0.0162	18.6	36.556	36.503
6	11.737	2	1	0	11.750	-0.0136	12.0	34.687	34.647
7	11.955	2	1	1	11.964	-0.0094	4.0	34.057	34.030
8	12.085	1	0	5	12.088	-0.0023	5.3	33.689	33.683
9	12.572	2	1	2	12.584	-0.0127	20.5	32.391	32.359
10	13.320	3	0	0	13.330	-0.0098	42.2	30.578	30.556
11	13.624	1	1	5	13.629	-0.0053	100.0	29.900	29.889
12	14.068	3	0	2	14.072	-0.0044	4.8	28.961	28.952
13	14.338	2	0	5	14.339	-0.0014	16.2	28.419	28.416
14	14.796	2	1	4	14.812	-0.0154	48.2	27.543	27.514
15	14.956	3	0	3	14.950	0.0066	9.8	27.250	27.262
16	15.543	1	1	6	15.549	-0.0058	30.6	26.227	26.217
		2	2	1	15.569	-0.0255			26.184
17	16.040	3	1	0	16.037	0.0033	37.6	25.419	25.424
		2	2	2	16.053	-0.0122			25.400
18	16.098	3	0	4	16.099	-0.0009	34.1	25.328	25.327
19	16.193	2	0	6	16.176	0.0170	19.0	25.180	25.207
		3	1	1	16.195	-0.0020			25.177
20	16.273	2	1	5	16.288	-0.0148	4.6	25.058	25.036
21	16.399	1	0	7	16.379	0.0203	5.3	24.866	24.897
22	16.653	3	1	2	16.662	-0.0083	8.1	24.490	24.477
23	16.828	2	2	3	16.829	-0.0013	12.9	24.237	24.236
24	17.403	3	1	3	17.412	-0.0092	5.1	23.443	23.431
25	17.542	1	1	7	17.555	-0.0132	7.9	23.259	23.241
26	17.827	4	0	0	17.805	0.0219	6.9	22.889	22.917
27	17.926	2	1	6	17.931	-0.0054	18.2	22.764	22.757
		4	0	1	17.948	-0.0226			22.736
28	19.419	3	2	0	19.417	0.0013	7.9	21.029	21.030
29	19.617	1	1	8	19.623	-0.0063	22.5	20.818	20.812

		3	1	5	19.626	-0.0093			20.809
30	20.145	2	0	8	20.128	0.0176	3.8	20.278	20.295
31	20.418	4	1	0	20.424	-0.0063	8.7	20.010	20.004
32	20.561	2	2	6	20.535	0.0260	12.2	19.872	19.897
		4	1	1	20.550	0.0109			19.883
		3	2	3	20.574	-0.0136			19.859
33	21.100	4	0	5	21.105	-0.0048	14.4	19.370	19.366
34	21.560	2	1	8	21.573	-0.0134	12.7	18.961	18.950
35	22.194	2	0	9	22.197	-0.0031	7.0	18.426	18.423
36	22.305	5	0	0	22.308	-0.0022	5.6	18.335	18.334
37	22.496	3	0	8	22.488	0.0078	17.3	18.182	18.188
		3	2	5	22.490	0.0051			18.186
38	22.591	0	0	10	22.589	0.0021	11.3	18.107	18.108
39	23.023	1	0	10	23.031	-0.0082	2.3	17.771	17.765
40	23.194	3	3	0	23.195	-0.0011	3.2	17.642	17.642
41	23.522	2	1	9	23.522	0.0002	9.0	17.399	17.399
42	23.649	4	2	0	23.626	0.0228	8.2	17.307	17.324
		3	3	2	23.637	0.0121			17.316
43	23.887	4	0	7	23.863	0.0236	14.2	17.137	17.154
		1	1	10	23.893	-0.0057			17.133
44	24.564	4	1	6	24.561	0.0024	20.1	16.672	16.674
45	24.887	5	1	0	24.879	0.0083	12.0	16.459	16.464
		0	0	11	24.882	0.0053			16.462
46	25.061	5	0	5	25.044	0.0165	10.0	16.347	16.357
47	25.581	2	2	9	25.586	-0.0052	7.0	16.020	16.016
48	25.844	3	3	5	25.843	0.0018	1.8	15.859	15.860
49	26.236	4	2	5	26.233	0.0024	5.2	15.626	15.628
50	26.844	6	0	0	26.845	-0.0014	4.0	15.279	15.278
51	27.189	0	0	12	27.185	0.0033	9.2	15.088	15.090
52	27.379	4	1	8	27.372	0.0068	14.6	14.986	14.989
		5	1	5	27.375	0.0046			14.988
53	27.458	5	0	7	27.431	0.0279	12.5	14.943	14.958
		2	2	10	27.456	0.0021			14.944
54	27.601	2	1	11	27.598	0.0034	8.1	14.867	14.869
		4	3	2	27.604	-0.0032			14.866
55	27.839	3	1	10	27.826	0.0131	11.0	14.743	14.750
56	28.238	3	2	9	28.239	-0.0009	5.6	14.539	14.538
57	28.728	4	3	4	28.722	0.0057	5.8	14.296	14.299
58	29.407	6	1	0	29.393	0.0142	6.6	13.973	13.979
		2	2	11	29.395	0.0116			13.978
		5	2	4	29.428	-0.0208			13.963
59	29.552	3	3	8	29.533	0.0193	8.4	13.906	13.915
		4	3	5	29.535	0.0172			13.914
60	29.857	1	0	13	29.847	0.0106	3.0	13.767	13.771
		4	2	8	29.879	-0.0214			13.757

61	30.168	6	0	6	30.161	0.0065	4.6	13.628	13.631
		6	1	3	30.189	-0.0212			13.619
62	30.520	4	3	6	30.501	0.0184	6.7	13.475	13.483
		1	1	13	30.529	-0.0094			13.471
63	31.155	5	2	6	31.170	-0.0152	2.0	13.206	13.200
		4	4	1	31.181	-0.0255			13.196
64	32.196	7	0	3	32.177	0.0191	4.1	12.790	12.798
65	32.522	3	0	13	32.498	0.0242	3.5	12.665	12.675
		6	0	8	32.527	-0.0054			12.663
66	32.914	4	2	10	32.916	-0.0026	5.0	12.519	12.518
67	33.461	2	2	13	33.443	0.0180	5.9	12.320	12.326
		5	2	8	33.472	-0.0109			12.316
		7	0	5	33.474	-0.0127			12.315
68	33.875	5	1	10	33.851	0.0233	7.3	12.174	12.182
		6	0	9	33.892	-0.0171			12.168
69	34.033	4	4	6	34.036	-0.0023	5.4	12.119	12.118
		2	1	14	34.036	-0.0027			12.118
		7	1	1	34.045	-0.0118			12.115
70	34.679	7	1	3	34.668	0.0105	5.3	11.900	11.903
		4	0	13	34.669	0.0100			11.903
		6	1	8	34.697	-0.0179			11.894
71	35.079	1	1	15	35.074	0.0050	4.1	11.768	11.770

2.3 $Cs_{2.27}K_{12.73}Tl_{27}$

Wavelength : 0.709300
Symmetry : Hexagonal P
Spacegroup : P -6 2 m
Refined cell parameters:
a = 10.203(7) Å
c = 16.800(9) Å
V = 1514.7(13) Å^3
Number of single indexed lines : 17
Number of unindexed lines : 0

N	2Th[obs]	H	K	L	2Th[calc]	obs-calc	Int.	d[obs]	d[calc]
1	2.399	0	0	1	2.419	-0.0202	1.4	169.416	168.003
2	4.859	0	0	2	4.839	0.02	33.6	83.657	84.001
3	5.192	1	0	1	5.198	-0.0066	41.1	78.304	78.205
4	8.320	1	1	1	8.333	-0.0122	5.3	48.887	48.815
5	10.798	1	1	3	10.792	0.006	4.7	37.692	37.713
6	11.744	2	0	3	11.737	0.0074	4.4	34.664	34.686
7	12.441	2	1	1	12.431	0.0101	1.8	32.730	32.757
8	13.144	2	1	2	13.124	0.0206	0.4	30.986	31.035
9	14.027	3	0	1	14.043	-0.0159	51.4	29.044	29.012
10	14.498	1	1	5	14.522	-0.0235	100	28.106	28.061
11	15.589	2	1	4	15.595	-0.0059	9.3	26.151	26.141
12	16.197	2	2	1	16.168	0.0286	11.6	25.175	25.219
13	16.628	1	1	6	16.615	0.0129	61.9	24.527	24.546
		3	1	0	16.641	-0.0137			24.507
14	16.975	0	0	7	16.995	-0.0202	13.2	24.029	24.000
15	19.022	2	1	6	19.027	-0.0056	7.6	21.463	21.457
16	24.395	0	0	10	24.373	0.0216	10.3	16.786	16.800
17	25.177	5	0	4	25.150	0.0267	1.1	16.272	16.289
		3	3	3	25.178	-0.0009			16.272
		4	0	7	25.206	-0.0292			16.254
18	25.584	4	2	3	25.608	-0.0245	16	16.018	16.003
19	25.682	1	1	10	25.682	-0.0002	15.1	15.957	15.957
	?	3	1	8	25.700	-0.0178			15.947
20	25.722 ?	3	1	8	25.700	0.0216	10.5	15.933	15.947
21	25.834	4	1	6	25.808	0.0262	5.4	15.865	15.881
		5	1	0	25.825	0.0087			15.870
22	26.222	5	0	5	26.211	0.0111	12	15.635	15.641
23	27.263	2	2	9	27.234	0.0294	4.7	15.048	15.064
		1	0	11	27.260	0.003			15.050
24	28.630	5	1	5	28.617	0.0134	2.5	14.344	14.350
		4	2	6	28.633	-0.0032			14.342

25	29.964	3	2	9	29.935	0.0295	0.5	13.719	13.732
		4	3	4	29.942	0.0225			13.729
		5	2	3	29.965	-0.001			13.718
		4	2	7	29.989	-0.0251			13.707
